# Sleep, Well-Being and Academic Performance: A Study in a Singapore Residential College

**DOI:** 10.3389/fpsyg.2021.672238

**Published:** 2021-05-31

**Authors:** Marc A. Armand, Federica Biassoni, Alberto Corrias

**Affiliations:** ^1^Department of Biomedical Engineering, National University of Singapore, Singapore, Singapore; ^2^Department of Psychology, Università Cattolica del Sacro Cuore, Milan, Italy; ^3^College of Alice and Peter Tan, National University of Singapore, Singapore, Singapore

**Keywords:** psychological well-being, positive/negative affect, overall sleep quality, academic performance, university students

## Abstract

We examined the relationship between sleep and the affective components of subjective well-being as well as psychological well-being, and between sleep and academic performance, of full-time undergraduate students in a residential college at the National University of Singapore. The aspects of sleep considered were self-reported sleep duration, sleep efficiency, frequency of sleep disturbances, daytime dysfunction, sleep latency and overall sleep quality, as measured by the Pittsburgh Sleep Quality Index. Academic performance was measured using self-reported cumulative average point scores, typically known as grade point average in other institutions. Psychological well-being and the affective components of subjective well-being were assessed using the Flourishing Scale and the Scale of Positive and Negative Experience, respectively. With the exception of sleep latency, our univariate analysis revealed significant associations between the abovementioned facets of sleep, and the affective components of subjective well-being. The analysis also revealed significant associations between the above sleep variables and psychological well-being, except sleep latency and frequency of sleep disturbances. Only daytime dysfunction was found to be significantly correlated with academic performance in our univariate analysis. In addition, our multivariate analysis shows that psychological well-being, affect balance and academic performance each has a direct effect on overall sleep quality. The relationship between overall sleep quality and psychological well-being is U-shaped, while that between overall sleep quality and affect balance is linear and moderated by psychological well-being. The relationship between overall sleep quality and academic performance is either U-shaped or an inverted-U, depending on the level of psychological well-being, which moderates the relationship. These nonlinear relationships indicate that individuals with the highest levels of psychological well-being are not the best sleepers (in terms of overall sleep quality), neither are the highest academic achievers necessarily the best sleepers.

## Introduction

It is known that sleep/wake timing shifts later due to pubertal changes of the circadian timing and homeostatic sleep systems during the second decade of life. Consequently, adolescents and young adults can experience sleep loss and excessive daytime sleepiness as they attempt to synchronize their natural delayed schedule with the requirements of everyday societal schedules such as school and office hours (Crowley et al., 2007; Alfonsi et al., 2020). University students living in halls of residence face additional challenges that can further affect their quality of sleep. Problems in their sleep environment may include noise and roommates’ different habits (Qin and Brown, 2017). The demands to contribute to the communal life of the hall and to integrate socially in its high-density living environment (Zhai et al., 2018), the stress from short-term academic workload and long-term anxiety related to independent adult life (Lemma et al., 2012; Laidlaw et al., 2016), and the lack of knowledge and practice of good sleep hygiene (Suen et al., 2010; Dinis and Braganca, 2018), further add to these problems. Not surprisingly, university students are viewed as being chronically sleep-deprived (Curcio et al., 2006; Fonseca and Genzel, 2020).

### Sleep and Well-Being

Since the 90’s, psychological research has highlighted the fundamental subjective nature of well-being. Subjective well-being is defined as “a person’s cognitive and affective evaluations of his or her life” (Diener et al., 2009) and comprises three components: satisfaction with life, presence of positive emotions and moods, and absence of negative emotions and moods. An individual is said to have high subjective well-being if she experiences high satisfaction with life, frequent positive affect and infrequent negative affect. Within the conceptual framework of well-being, the idea of psychological well-being emerged as a state in which an individual realizes her own potential, relating to the social and physical environment satisfactorily and being able to cope with stressors (Ryan and Deci, 2001; Keyes et al., 2002; Hernandez-Torrano et al., 2020). Given the U-shaped relationship between sleep duration and morbidity and mortality established in epidemiological studies (Ikehara et al., 2009; Kronholm et al., 2011; Li et al., 2015), a nonlinear relationship between sleep and well-being is expected (Hamilton et al., 2007b).

Richter (2015) found sleep deprivation to be negatively correlated with psychological well-being within a university student population. Zhai et al. (2018), taking into exam the role of sleep quality in the psychological well-being of final year undergraduate students, reported that poor sleep quality is associated with high levels of negative psychological well-being. Similarly, in a study involving subjects of age 18 and above (the majority being students), Freitag et al. (2017) found that sleep disturbances were related to decreased levels of psychological well-being. In addition, from a large sample of university students from 16 countries, Allgower et al. (2001) found that excessive (>9 h) or insufficient (<7 h) sleep was linked to increased risk in social isolation, thus implying an inverted U-shaped relationship between psychological well-being and sleep duration. Several studies have found sleep quantity and/or quality to be related to the affective components of subjective well-being. For example, Pilcher et al. (1997) found that average sleep quality was better related to affect balance than average sleep quantity among college students who slept an average of 7 h a night. Lemma et al. (2012) and Lund et al. (2010) found poor sleep quality among university students to be associated with higher degrees of negative affect including anger, confusion, depression and tension. Similarly, Li et al. (2020) found that both poor sleep quality and insufficient sleep were associated with depression in university students. Fulgini and Hardway (2006) found that shorter sleep was associated with more negative and less positive moods. Interestingly, Lima et al. (2018) reported a U-shaped relationship between happiness–which constitutes positive affect–and sleep duration in adults aged 20 and older, with a prevalence of unhappy individuals among short (≤6 h) and excessively long (≥9 h) sleepers.

### Sleep and Academic Achievement

In the last decade, a number of studies on the interaction between subjective sleep and academic achievement of university students have emerged. Ahrberg et al. (2012), Baert et al. (2015), and Toscano-Hermoso et al. (2020) reported a positive relationship between sleep quality and academic scores. Gomes et al. (2011) found poor sleep quality and insufficient sleep to be significantly associated with poorer academic performance. Zeek et al. (2015) and Raley et al. (2016) similarly found that longer sleep the night prior to an examination was associated with higher course grades. Academic performance has also been found to be negatively correlated with sleep latency (Chiang et al., 2014; Leak et al., 2020). In addition, Datta et al. (2019) reported a substantially higher proportion of students with disturbed sleep among those with average exam marks compared to students with good marks. Similarly, Maheshwari and Shaukat (2019) found a negative association between frequency of sleep disturbances and GPA scores. They also reported that most students with low GPAs had sleep efficiencies of only 75–84% and experienced daytime dysfunction almost every day. Departing from these studies, Okano et al. (2019) found that both objective sleep quantity and quality for the month and week before a test positively correlated with academic grades.

There are nevertheless studies that report contrary findings. For example, Dokuka and Smirnov (2020) found high academic performance to be associated with shorter sleep among young adults between 20 and 21 years of age including university students. Also, Hangouche et al. (2018) did not find excessive daytime sleepiness (i.e., uncontrollable dozing off and drowsiness during the daytime) to be related to academic performance. At the other end of the spectrum, a few studies have reported no association between sleep and academic achievement. For example, Sweileh et al. (2011) and Jalali et al. (2020) found no significant difference in sleep quality between students with high grades and those with low grades. In addition, Eliasson et al. (2010) found that total sleep duration (from daytime naps plus nocturnal sleep) does not correlate with academic performance. In another objective sleep study, King et al. (2018) reported no difference in project grades between students who averaged at least 8 h of sleep for five nights leading up to the project’s due date and those who did not. In short, recent findings reported in the literature on the relationship between sleep and academic performance have not fully converged to a consensus, as noted by Jalali et al. (2020).

### The Present Study

The present study had two main objectives. The first was to understand how psychological well-being, the affective components of subjective well-being, and academic performance are related to self-reported sleep of full-time undergraduate students residing at the College of Alice and Peter Tan (CAPT) at the National University of Singapore. The targeted endpoint was a model for subjective sleep quality that would reveal the nature of these relationships (e.g., linear versus quadratic) and how the different dependent variables might interact. The second objective was to compare how CAPT students fair compared to university students in other countries in terms of subjective sleep quality as well as psychological and affective well-being.

## Materials and Methods

### Instruments

The Pittsburgh Sleep Quality Index (PSQI; Buysse et al., 1989) is aself-rated questionnaire which assesses sleep quality anddisturbances over a 1-month time interval. It measures sevendimensions of sleep: subjective sleep quality, sleep latency, sleep duration, sleep efficiency, frequency of various sleep disturbances, frequency of usage of sleep medication, and frequency and severity of daytime dysfunction. Each subscale is scored on a 4-point scale ranging from 0 to 3. Summing the score of all seven subscales yields the Global PSQI score which represents overall sleep quality. The Global PSQI score therefore ranges between 0 and 21. A higher Global PSQI indicates poorer overall sleep quality. A higher score for each subscale is likewise a poorer score. An individual with a Global PSQI score greater than 5 is viewed to be a poor sleeper (Buysse et al., 1989). Conversely, an individual with a Global PSQI score not exceeding 5 is considered a good sleeper. The PSQI questionnaire consists of 10 questions of which Q.1–Q.9 are designed for self-assessment while Q.10 is to be completed by a roommate or bed partner, based on sleeping patterns over the past month. The rooms at the residential college considered in this study are all individual rooms and, as such, Q.10 was not scored, and only Q.1–Q.9 were used in our survey.

In the PSQI scoring procedure, the scores assigned to Q.5b–Q.5j are added together and the sum, which ranges between 0 and 27, is then mapped to a final score ranging between 0 and 3 to represent the frequency of sleep disturbances. Since this mapping causes us to lose the granularity of the information inherent in the original sum (e.g., distinct sums such as two and eight become indistinguishable once mapped to one), we used the sum and not the final score as the measure of this sleep dimension. Larger sums represent higher frequencies of sleep disturbances. For the same reason, we used the sum of scores assigned to Q.8 and Q.9 and not the final score derived from the sum, to represent the extent of daytime dysfunction experienced. Similarly, a larger sum represents more frequent and/or more severe daytime dysfunction.

The Scale of Positive and Negative Experience (SPANE; Diener et al., 2010) is a 12-item questionnaire designed to assess subjective feelings ofwell-being and ill-being, that is to measure the affective componentsof well-being. Six items measure the frequency of experiencing arange of positive feelings over the past four weeks, while the other six measure the frequency of experiencing a range of negative feelings over the same period. Each item is scored using a 5-point scale ranging from 1 to 5, with larger scores representing higher frequencies. Summing the scores for the six items measuring positive (respectively, negative) affect yields the overall positive (respectively, negative) affect score, denoted SPANE-P (respectively, SPANE-N), which ranges between 6 and 30. Larger SPANE-P (respectively, SPANE-N) scores represent higher frequencies of experiencing positive (respectively, negative) affect. Subtracting SPANE-N from SPANE-P yields the affect balance score, denoted SPANE-B, which ranges between -24 and +24. Increasingly positive (respectively, negative) SPANE-B scores represent increasingly higher (respectively, lower) frequencies of experiencing positive affect compared to negative affect. For both the positive and negative items, three of the items are general (e.g., positive and negative) and three per subscale are more specific (e.g., joyful and sad). Because of the general items included in the scale, it can assess not only the pleasant and unpleasant emotional feelings that are the focus of most scales, but also reflect other states such as interest, flow, positive engagement, and physical pleasure.

The Flourishing Scale (FS; Diener et al., 2010), is a brief 8-item summarymeasure of the respondent’s self-perceived success in important areassuch as relationships, self-esteem, purpose, and optimism. The scaleprovides a single psychological well-being score. The surveycomprises several items on satisfaction with social relationships (having supportive and rewarding relationships, contributing to the happiness of others, and being respected by others), an item on having a purposeful and meaningful life, and one on being engaged and interested in one’s activities, and one on feeling competent and capable in the activities that are important to the respondent. Finally two items are included tapping self-respect and optimism. Thus, FS assesses major aspects of social–psychological functioning from the respondent’s own point of view. Each item is scored using a 7-point scale ranging from 1 to 7 to represent strongly disagree to strongly agree. Summing the scores yields the (overall) FS score which ranges between 8 and 56. An individual with a high FS score has many psychological resources and strengths (Diener et al., 2010).

### Participants and Bad Data Points

As previously mentioned, the subjects of our study were full-time undergraduate students from the College of Alice and Peter Tan (CAPT), a residential college within the National University of Singapore. In any given semester, the college is home to approximately 500 students. The gender ratio in the population is close to 50–50 with a slight predominance of female students. The age range of female students is 18–22 while that of male students is 18–24. The wider age range among male students is due to the national service obligations that Singaporean males have to fulfill prior to entering university. In total, 144 students participated in the survey. Demographic data, such as age and gender, was not captured in the survey.

Upon inspecting the completed questionnaires, we found meaninglessresponses from four participants pertaining to Q.1–Q.4 in the PSQI questionnaire, which we believe to be mainly due to the open-ended nature of these four questions. Two of these participants answered “all the time” and “Yes” in response to Q.3 instead of stating their typical waking times, thus rendering the data they provided incomplete. The other two participants provided answers to Q.1, Q.2, and Q.4 that resulted in sleep efficiencies (i.e., the percentage of hours slept of the total time in bed) that did not make sense, such as 133%. These four participants were therefore removed from our sample, reducing our sample size to 140.

Note, however, that not all the participants had a CAP at the time the survey was conducted. This is due to the fact that some respondents were freshmen in their first semester at NUS and therefore had not yet obtained any grade. Consequently, for analyses involving academic performance, the sample size had to be further reduced to 106.

### Statistical Methods

In our univariate analyses, we examined the strength of association between sleep and psychological well-being, as well as between sleep and CAP scores, using the Spearman rank correlation coefficient as well as the Kendall rank correlation coefficient (As both correlation measures resulted in the same set of statistically significant associations, we report our findings in terms of the former measure for brevity). We did not use the Pearson product-moment correlation coefficient for cases where the variables were both continuous to take into account the possibility of nonlinear relationships between such variables.

For our multivariate analysis, we used moderated linear regression to model overall sleep quality. The dependent variable is Global PSQI while the linear terms among the independent variables are SPANE-B, FS, and CAP. As we believed that the relationship between overall sleep quality, and SPANE-B, FS, and CAP, could be nonlinear, we included quadratic terms SPANE-B^2^, FS^2^, and CAP^2^. As we also believed that these 6 explanatory variables may interact, the following second and third order product terms were included: SPANE-B × FS, SPANE-B × FS^2^, SPANE-B^2^ × FS, CAP × SPANE-B, CAP × SPANE-B^2^, CAP^2^ × SPANE-B, CAP × FS, CAP × FS^2^, and CAP^2^ × FS. Consequently, we had a total of 15 explanatory variables to begin with. To reduce multicollinearity, we adopted the standard practice of mean-centering the linear terms (Iacobucci et al., 2016). For example, each FS score was transformed by subtracting the sample mean *m*_*FS*_ of the FS scores. Squaring the transformed linear terms then yielded the corresponding quadratic terms. From the six transformed linear and quadratic terms, the corresponding second and third-order product terms were then generated. The resulting transformed variables are distinguished from the original ones by adding the prefix “c” to each variable’s label. For instance, the transformed versions of FS, FS^2^, and FS^2^ × CAP are denoted cFS, cFS^2^, and cFS^2^ × cCAP, respectively. Finally, backward elimination was applied on the transformed explanatory variables. That is, starting with all 15 of them, the least significant variable was discarded one by one until only significant variables remain–a variable is viewed to be insignificant if its *p*-value is 0.05 or greater.

To compare the sleep quality and well-being of CAPT students to that of other student populations, we used a two-tailed Welch’s *t*-test to conduct two (independent) sample means tests with unequal variance. The sample means involved in these tests were the mean SPANE-P, SPANE-N, FS, and Global PSQI scores. Even though the frequency distributions of these variables are skewed, there are sound reasons to use Welch’s *t*-test instead of a non-parametric test. Firstly, due to the Central Limit Theorem, the *t*-test is increasingly robust to deviations from normality as sample size increases, and is robust even to heavily skewed distributions when the sample size is 200 (Fagerland and Sandvik, 2009). In addition, Fagerland (2012) compared the rejection rates of the Wilcoxon rank sum test and Welch’s *t*-test for samples drawn from pairs of Gamma and lognormal distributions of increasing sizes. For both distribution types, it was observed that the rejection rate of the Wilcoxon rank sum test increases rapidly with sample size whereas the rejection rate of the *t*-test remains stable at roughly the expected rejection rate of an unbiased test. Consequently, Fagerland (2012) asserts that non-parametric tests are most useful for small studies but for large sample sizes, *t*-tests should be used, even for heavily skewed data. Lumley et al. (2002) similarly showed that the *t*-test can perform well in moderately large sample sizes even for very non-normal distributions.

### Procedure

College of Alice and Peter Tan students were invited via email toparticipate in an online survey comprising the above threeinstruments plus a question to indicate their CAP score if they hadone. The online survey was accessible to students for a period ofthree weeks, during which they could complete the different questionnaires at their own pace. To mitigate the risk that students do not complete the survey within the 3-weeks time frame, several student leaders within CAPT were engaged to help promote the survey among their peers. Each participant was given a $5 supermarket voucher as a token of appreciation for completing the survey.

In particular, they received information that their participation in the present study was voluntary and that they could withdraw at any moment without any consequence in which case, their collected data would be discarded immediately. The study obtained the approval of the National University of Singapore’s Institutional Review Board.

## Results

### The Relationship Between Sleep and Well-Being/Academic Performance

Recall, the different aspects of sleep considered were (i) overall sleep quality, i.e., the Global PSQI score, (ii) sleep duration (in hours), (iii) sleep efficiency, (iv) frequency of sleep disturbances, (v) daytime dysfunction, and (vi) sleep latency (in minutes). Table 1 summarizes the resulting correlation coefficients and corresponding *p*-values (two-tailed).

**TABLE 1:** Spearman’s correlation between different sleep and well-being measures and the corresponding *p*-values.

	Global PSQI	Sleep Duration	Sleep Efficiency	Freq. of Sleep Disturbance	Daytime Dysfunction	Sleep Latency
SPANE-P	−0.441 (4.96E-8**)	0.189 (0.025*)	0.245 (3.46E-3**)	−0.211 (0.012*)	−0.354 (1.75E-5**)	−0.046 (0.587)
SPANE-N	0.348 (2.55E-5**)	−0.120 (0.157)	−0.078 (0.359)	0.190 (0.025*)	0.384 (2.85E-6**)	0.044 (0.607)
SPANE-B	−0.454 (1.71E-8**)	0.169 (0.046*)	0.176 (0.038*)	−0.232 (5.76E-3**)	−0.421 (2.25E-7**)	−0.073 (0.388)
FS	−0.374 (5.24E-6**)	0.253 (2.53E-3**)	0.177 (0.037*)	−0.074 (0.388)	−0.291 (4.91E-4**)	−0.047 (0.581)
CAP	−0.114 (0.245)	−0.019 (0.85)	−0.082 (0.402)	−0.156 (0.111)	−0.240 (0.013*)	−0.166 (0.089)

*Denotes p < 0.05. **Denotes p < 0.01.

Both Global PSQI and daytime dysfunction correlate moderately with the four well-being measures (All eight correlation indices are significant at the 0.01 level). Sleep duration and sleep efficiency, on the other hand, correlate weakly with SPANE-P, SPANE-B, and FS (All eight correlation indices in this case are significant at the 0.05 level or lower). Similarly, the correlation indices of frequency of sleep disturbance and SPANE-P, SPANE-N, and SPANE-B are weak but statistically significant at the 0.05 level or lower. Sleep duration and sleep efficiency, and frequency of sleep disturbance, do not appear to be related to SPANE-N and FS, respectively.

Turning to academic performance, we see that daytime dysfunction correlates weakly with CAP scores (The correlation index is significant at the 0.05 level). There is, however, insufficient evidence of any association between CAP scores and the other sleep variables including Global PSQI.

### A Model for Overall Sleep Quality

In this section, we present a model–called the *full* model–for overall sleep quality obtained through moderated linear regression. Table 2 summarizes the results of the final iteration of the backward elimination procedure. From this table, we have the following regression equation:

$$\begin{array}{c} \mathrm{Global}\mathrm{PSQI}=b_{0}+b_{2}{\text{cFS}}^{2}+(b_{1}+b_{4}\text{cFS}\\ \;+b_{5}{\text{cFS}}^{2})\text{cSPANE-B}+(b_{3}+b_{6}\text{cFS})\text{cCAP}{}^{2}. \end{array}$$

**TABLE 2:** Effects of the Scale of Positive and Negative Experience (SPANE)-B, Flourishing Scale (FS), and Cumulative Average Point (CAP) on Global Pittsburgh Sleep Quality Index (PSQI) in the full, reduced, and reduced-plus models.

Full model					

Independent variables		Slope, *b*_i_	Std. error	*t*-statistic	*p*-value
(0) Constant		6.608			
(1) cSPANE-B		−0.148	0.032	−4.704	8.27E-6**
(2) cFS^2^		−2.62E-3	1.22E-3	−2.147	0.034*
(3) cCAP^2^		2.369	0.755	3.139	2.24E-3**
(4) cFS × cSPANE-B		5.11E-3	2.06E-3	2.484	0.015*
(5) cFS^2^ × cSPANE-B		2.66E-4	1.33E-4	1.992	0.049*
(6) cFS × cCAP^2^		−0.532	0.118	−4.490	1.93E-5**
*R*^2^	=0.411				
Std. Error	=1.787				
*F*-statistic	=11.508				
*p*-value of *F*	=9.80E-10**				

**Reduced model**					

**Independent variables**		**Slope**	**Std. error**	***t*-statistic**	***p*-value**

(0) Constant		6.535			
(1) cSPANE-B		−0.122	0.024	−5.081	1.70E-6**
(2) cFS^2^		3.03E-5	0.001	0.030	0.976
(3) cCAP^2^		1.854	0.835	2.221	0.029*
*R*^2^	=0.241				
Std. Error	=1.998				
*F*-statistic	=10.817				
*p*-value of *F*	=3.13E-6**				

**Reduced-plus model**					

**Independent variables**		**Slope**	**Std. error**	***t*-statistic**	***p*-value**

(0) Constant		7.021			
(1) cSPANE-B		−0.073	0.026	–2.796	6.19E-3**
(2) cFS		−0.098	0.026	−3.761	2.84E-4**
(3) cFS^2^		−3.96E-3	1.42E-3	–2.788	6.34E-3**
(4) cCAP^2^		1.708	0.787	2.172	0.032*
*R*^2^	=0.335				
Std. Error	=1.881				
*F*-statistic	=12.693				
*p*-value of *F*	=2.09E–8**				

*Denotes p < 0.05. **Denotes p < 0.01.

The left-hand side of the equation should be understood to mean the fitted value of Global PSQI for a given set of values for cFS, cSPANE-B, and cCAP. Evidently, all six explanatory variables in the equation are significant. We see that psychological well-being is quadratically related to overall sleep quality, after controlling for the other variables in the model, i.e., when the other variables–in their original form–assume their respective mean values. In addition, there is a linear relationship between overall sleep quality and affect balance, after controlling for the other variables in the model, and this relationship is moderated by psychological well-being. Further, there is a quadratic relationship between overall sleep quality and academic performance, after controlling for the other variables, and this relationship is also moderated by psychological well-being. The constant, *b*_0_, is the expected value of Global PSQI when SPANE-B, FS, and CAP assume their mean values of 4.264, 41.123, and 4.269, respectively.

**FIGURE 1:**
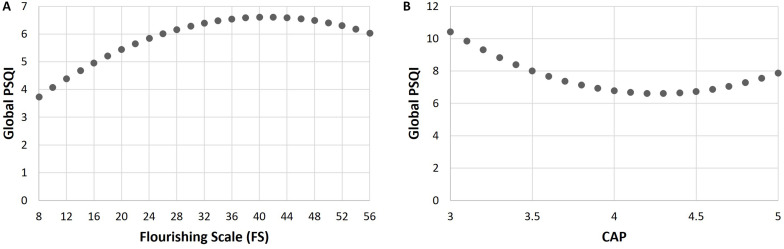
Relationship between global Pittsburgh Sleep Quality Index (PSQI) and Flourishing Scale (FS) scores in panel **(A)**, and that between Global PSQI and Cumulative Average Point (CAP) scores in panel **(B)**, after controlling for the other variables in the full model.

The abovementioned quadratic relationship between Global PSQI and FS, which is inverted U-shaped, is shown in Figure 1A, while that between Global PSQI and CAP is shown in Figure 1B. The turning points of these curves coincide with the mean FS score and mean CAP score, respectively. Although the latter relationship is depicted as being U-shaped, note that this is not always the case. One readily checks that the sign of the coefficient (*b*_3_ + *b*_6_cFS) of cCAP^2^ in the above regression equation is negative for FS scores 46 and above (i.e., ≥*m*_FS_ + 0.45*s*_FS_ where *s*_*FS*_ denotes the standard deviation of the FS scores), and positive otherwise.

If we remove the interaction terms from our full model (other than the quadratic terms) to obtain a *reduced* model, the effects of the remaining independent variables on overall sleep quality are as shown in Table 2. The change in *R*^2^ between the full and reduced model is 0.170, for which the associated *p*-value is 1.43E-5. This provides evidence that there is significant interaction between psychological well-being and affect balance, and between psychological well-being and academic performance.

The slope corresponding to cFS^2^ in the reduced model is nevertheless not significant. Beginning with cSPANE-B, cFS, cCAP, cSPANE-B^2^, cFS^2^, and cCAP^2^ and applying backward elimination, however, yields a model that also contains the three independent variables in the reduced model, plus the linear term, cFS. Table 2 summarizes the effects of these four variables on overall sleep quality in this model, which we refer to as the *reduced-plus* model. The change in *R*^2^ between the full model and the reduced-plus model is 0.076, for which the corresponding *p*-value is 2.40E-3. The full model therefore provides a significant improvement over the reduced-plus model in the quality of fit as measured by *R*^2^.

**FIGURE 2:**
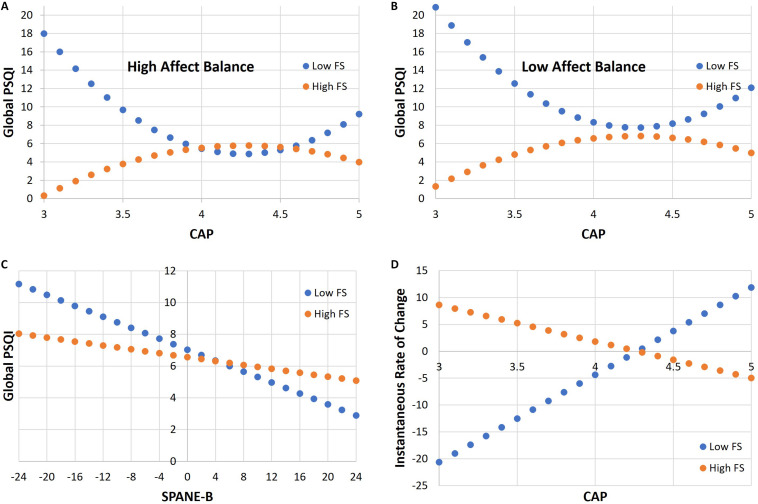
Interaction plots of Global PSQI versus cumulative average point (CAP) scores for high affect balance in panel **(A)**; Interaction plots of Global PSQI versus CAP scores for low affect balance in panel **(B)**; Interaction plot of Global PSQI versus Scale of Positive and Negative Experience (SPANE)-B scores in panel **(C)**; Instantaneous rate of change in Global PSQI for low and high levels of psychological well-being in panel **(D)**.

A visualization of the contrasting effects on overall sleep quality at low and high levels of psychological well-being in our full model is provided by the interaction plots in Figures 2A,B. The plots in Panel A (respectively, B) correspond to SPANE-B = *m*_SB_ + *s*_SB_ = 12.596 (respectively, SPANE-B = *m*_SB_−*s*_SB_ = −4.067) where *m*_*SB*_ and *s*_*SB*_ denote the mean and standard deviation of SPANE-B scores, respectively. Similarly, we take FS = *m*_FS_ + *s*_FS_ = 51.967 and FS = *m*_FS_−*s*_FS_ = 30.279 to represent high and low levels of psychological well-being, and refer to these values as *high* FS and *low* FS, respectively.

The linear relationship between Global PSQI and SPANE-B is negative with a steeper (respectively, gentler) slope at low (respectively, high) levels of psychological well-being, as depicted by the interaction plot in Figure 2C for which CAP is held constant at the mean. At low (respectively, high) FS, the slope is $b_{1}-b_{4}s_{\text{FS}}+b_{5}s_{\text{FS}}^{2}=-0.172$ (respectively, $b_{1}+b_{4}s_{\text{FS}}+b_{5}s_{\text{FS}}^{2}=-0.062$). In other words, the expected increase in Global PSQI for a unit decrease in SPANE-B is 0.17 at low FS and 0.06 at high FS. A drop from high to low affect balance therefore translates to an expected increase in Global PSQI of 2.87 at low FS and 1.03 at high FS.

Finally, observe that for a given CAP score (other than the mean CAP), the instantaneous rate of change in overall sleep quality differs at low and high levels of psychological well-being. It is specified by the partial derivative of the regression equation with respect to cCAP, which by the chain rule, can be rewritten as

$$\frac{d\mathrm{Global}\mathrm{PSQI}}{d\text{CAP}}=2\left(b_{3}+b_{6}\text{cFS}\right)\text{cCAP}.$$

Figure 2D shows the instantaneous rate of change for low and high levels of psychological well-being.

The expected change in Global PSQI for a unit increase in CAP is then given by:

$$\begin{array}{c} \frac{2\left(b_{3}+b_{6}\text{cFS}\right)\text{cCAP}+2\left(b_{3}+b_{6}\text{cFS}\right)\left(\text{cCAP}+1\right)}{2}\\ \;\;=\left(b_{3}+b_{6}\text{cFS}\right)\left(2\text{cCAP}+1\right). \end{array}$$

Similarly, the expected change in Global PSQI for a unit decrease in CAP is

$$\left(b_{3}+b_{6}\text{cFS}\right)\left(2\text{cCAP}-1\right).$$

For example, at low FS, the expected change in Global PSQI corresponding to an increase in CAP from 3.0 to 4.0 is (*b*_3_−*b*_6_*s*_FS_)(2×(3−*m*_CAP_) + 1) = −12.52 where *m*_*CAP*_ denotes the mean CAP. At high FS, however, the expected change is (*b*_3_ + *b*_6_*s*_FS_)(2×(3−*m*_CAP_) + 1) = 5.23.

### Global PSQI, SPANE, and FS Scores of Other Student Populations

The overall sample mean, SD, median and interquartile range (IQR) ofthe Global PSQI, SPANE, and FS scores for the 140 CAPT students surveyed are presented in Table 3. For the purpose of comparison, we present in Table 4 the mean and standard deviation of the SPANE-P and SPANE-N scores of *n* university students in seven countries: United States (Diener et al., 2010), Canada (Howell and Buro, 2015), Germany (Rahm et al., 2017), Portugal (Silva and Caetano, 2013), Japan (Sumi, 2014), Singapore (Diener et al., 2010), and South Africa (du Plessis and Guse, 2016). The students in Singapore were from the Singapore Management University (SMU). Where available, we also include in Table 4 the mean and standard deviation of the FS scores of these student populations.

**TABLE 3:** Descriptive statistics of Global Pittsburgh Sleep Quality Index (PSQI), Scale of Positive and Negative Experience (SPANE), and Flourishing Scale (FS) scores for College of Alice and Peter Tan (CAPT) students.

	Mean (SD)	Median (IQR)
Global PSQI	6.86 (2.25)	7 (5–8)
SPANE-P	21.17 (4.60)	22 (18–24)
SPANE-N	16.89 (4.49)	17 (14–20)
SPANE-B	4.28 (8.08)	4.5 (-1–10)
FS	41.58 (10.12)	44 (38–48)

**TABLE 4:** Mean and standard deviation of Scale of Positive and Negative Experience (SPANE) and Flourishing Scale (FS) scores of other university student populations.

	*n*	SPANE-P Mean (SD)	SPANE-N Mean (SD)	FS Mean (SD)	SPANE-P *p*-value	SPANE-N *p*-value	FS *p*-value
United States	168	23.10 (3.20)	14.50 (3.60)	48.10 (4.90)	3.90E-5**	7.00E-7**	ε**
Canada	478	22.49 (4.08)	15.78 (4.07)	46.69 (6.73)	2.50E-3**	9.27E-3**	8.00E-8**
Germany	498	22.25 (3.88)	14.31 (4.24)		0.012*	ε**	
Portugal	194	23.51 (4.03)	13.30 (4.66)	44.51 (4.86)	2.28E-6**	ε**	1.77E-3**
Japan	520	21.01 (4.50)	16.61 (4.87)	36.63 (8.05)	0.714	0.521	2.60E-7**
Singapore	181	20.80 (3.60)	17.00 (4.00)	42.60 (6.40)	0.434	0.820	0.299
South Africa	992	21.91 (3.81)	15.96 (3.94)		0.071	0.021*	

*Denotes p < 0.05. **Denotes p < 0.01.

The last 3 columns of Table 4 summarizes the *p*-values resulting from the application of Welch’s *t*-test to compare the means presented in Table 3 and their counterparts in Table 4 (Note that infinitesimally small *p*-values are denoted by ε). For SPANE-P scores, the differences in the mean between CAPT students and students in United States, Canada, Germany and Portugal are significant at the 0.05 level or lower. On the other hand, the differences are not significant when comparing with students in Japan, South Africa, and not surprisingly, SMU. For SPANE-N scores, only the differences in the mean between CAPT students and students in Japan and SMU are not significant.

Finally, to determine if CAPT students also have poorer overall sleep quality compared to student populations elsewhere, we summarize in Table 5 the mean and standard deviation of the Global PSQI scores of *n* university students in eight countries: United States (Carney et al., 2006), Brazil (Mesquita and Reimao, 2010), Belgium (Baert et al., 2015), Luxembourg and Germany (Schlarb et al., 2017), Taiwan (Cheng et al., 2012), Indonesia (Herawati and Gayatri, 2019), and Nigeria (Aloba et al., 2007). For easy visual comparison, the last row of Table 5 presents the corresponding numbers for CAPT students. The last column of the table presents the *p*-values resulting from the application of Welch’s *t*-test to compare the mean Global PSQI score of CAPT students and that of these other student populations. Evidently, only the differences in the means between CAPT students and students in Brazil, and Luxembourg and Germany are not significant.

**TABLE 5:** Mean and standard deviation of Global Pittsburgh Sleep Quality Index (PSQI) scores of other university student populations.

	*n*	Global PSQI Mean (SD)	*p*-value
United States	243	5.6 (3.4)	1.73E-5**
Brazil	710	6.5 (2.6)	0.094
Belgium	621	4.802 (2.228)	ε**
Luxembourg and Germany	2831	7.22 (3.70)	0.077
Taiwan	4318	6.0 (2.5)	1.77E-5**
Indonesia	450	8.40 (3.64)	ε**
Nigeria	520	4.43 (2.67)	ε**
This study	140	6.86 (2.25)	

**Denotes p < 0.01.

## Discussion

In the following discussion, it is convenient to interpret sleep duration as sleep *quantity*, and sleep efficiency, frequency of sleep disturbance, daytime dysfunction and sleep latency as individual measures of sleep *quality* (since they contribute to the overall sleep quality measure, i.e., the Global PSQI score). Also, since higher Global PSQI scores represent poorer overall sleep quality, the direction of the relationships presented in Section “A Model for Overall Sleep Quality” involving Global PSQI will have to be flipped when speaking in terms of overall sleep quality.

### Reconciling Inconsistency Between the Univariate and Multivariate Analyses

**FIGURE 3:**
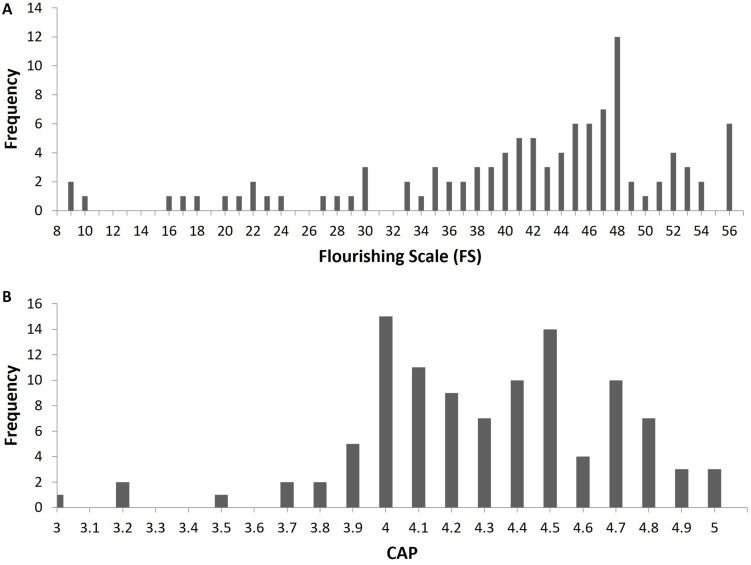
Frequency distribution of Flourishing Scale (FS) scores in panel **(A)**; frequency distribution of Cumulative Average Point (CAP) scores in panel **(B)**.

It is interesting to note that despite Global PSQI and FS having a non-monotonic relationship as revealed by our multivariate analysis, we obtained a significant, negative Spearman correlation between the two variables, as reported in Table 1. We believe that this is due to the frequency distribution of FS scores which has a skew of -1.15 and so is highly left-skewed. In other words, the left-tail of the distribution is very long and thin, with the majority of the values falling to the right of the mean, as shown in Figure 3A. The relationship between Global PSQI and CAP is likewise non-monotonic. However, Spearman’s correlation between Global PSQI and CAP is not significant, as highlighted in Section “The Relationship Between Sleep and Well-Being/Academic Performance”. We believe that there are two main reasons. Firstly, the frequency distribution of CAP scores is apparently bimodal with one mode falling on each side of the mean, which implies that both the left and right shoulders of the distribution are pronounced, as shown in Figure 3B. Secondly, and as a consequence of the first reason, the distribution has a skew of -0.58 and therefore is only moderately left-skewed.

### Sleep and Psychological Well-Being

Contrary to the findings of Freitag et al. (2017), our univariate analysis did not reveal any relationship between frequency of sleep disturbances and psychological well-being. Our analysis, however, revealed that sleep duration has a positive association with psychological well-being, a result that is consistent with the findings of Richter (2015). Similarly, just as Zhai et al. (2018) found a strong relationship between sleep quality and psychological well-being, our univariate analysis showed that sleep efficiency and overall sleep quality are significantly and positively correlated with the same.

Our multivariate analysis nevertheless informs that the positive association between overall sleep quality and psychological well-being is only true for above average levels of the latter. Specifically, it showed that psychological well-being has a direct, U-shaped quadratic relationship with overall sleep quality. The turning point of this quadratic relationship coincides with the mean level of psychological well-being. Thus, for above (respectively, below) average levels of psychological well-being, overall sleep quality improves with increasing (respectively, decreasing) levels of psychological well-being. This curvilinear relationship suggests that attaining increasingly greater psychosocial prosperity up to a certain point comes at the price of deteriorating overall sleep quality. Beyond that point, however, overall sleep quality starts to improve, meaning that above average levels of psychological well-being may be viewed as a resource for regulating overall sleep quality.

To provide a speculative explanation for this curvilinear relationship, we tap on recent findings pertaining to the relationship between perceived stress and sleep quality. Firstly, resilience has been found to be highly and positively correlated with psychological well-being among university students (Pidgeon and Keye, 2014). Secondly, it moderates (i.e., weakens) the relationship between perceived stress and sleep quality (Li et al., 2019; Du et al., 2020). We also extend the notion of sleep quality affording a protective value on subjective well-being (Weinberg et al., 2016), to psychological well-being. Finally, we make use of the fact that among the Big Five personality traits, neuroticism has been consistently found to be related to poorer psychological well-being (Brown and Ryan, 2003).

With that, we hypothesize that as an individual with poor psychological well-being starts to strengthen or expand her network of social relationships, find more purpose and meaning in her life and so on, the perceived stress engendered in the process, grows in intensity. Her level of resilience at this stage is, however, too low to negate the negative effects of the perceived stress on her overall sleep quality. On the other hand, the decrease in sleep quality is not enough to suppress the protective value it affords on her psychological well-being, thus enabling her to perceive an elevated level of the same. This trend of deteriorating overall sleep quality (but improving psychological well-being) continues until a certain level of psychological well-being unique to the individual is reached. At this point, the individual’s increased level of resilience is sufficiently high to negate the effects of the perceived stress on sleep. This paves the way for the trend to reverse, with the achieved higher level of psychological well-being now serving to promote better sleep (Steptoe et al., 2008).

We expect this reversal to take place gradually, since Figure 1A shows an inverted “U” rather than an inverted “V”. In other words, the transition from overall sleep quality protecting psychological well-being, to the latter promoting the former, is a gradual process.

### Sleep and the Affective Components of Subjective Well-Being

Our univariate analysis revealed that sleep quantity and the aforementioned sleep quality measures correlated significantly with positive affect and affect balance, with the exception of sleep latency which did not appear to have any association with the affective components of subjective well-being. These results are consistent with the findings of other studies, for instance, Steptoe et al. (2008), which reported a negative association between sleep problems and positive affect, Pilcher et al. (1997), which found both sleep quantity and quality to be related to affect balance, and (Fulgini and Hardway, 2006), which found shorter sleep to be associated with less positive moods. Congruent with findings by Lund et al. (2010), Lemma et al. (2012), and Li et al. (2020), we also found that an increase in negative affect significantly correlates with increasing frequency of sleep disturbance and daytime dysfunction. Overall, these results are consistent with the theoretical model due to Zohar et al. (2005) and Hamilton et al. (2007a) in which sleep is a resource for regulating emotional responses to goal-disruptive/-enhancing events including stressful situations.

Adding to the literature that affect balance is a resource forregulating overall sleep quality, our multivariate analysis further showed that overall sleep quality decreases linearly with affect balance, and that this relationship is moderated by psychological well-being; at lower (respectively, higher) levels of psychological well-being, the decline in overall sleep quality with affect balance is faster (respectively, slower). To provide a speculative explanation for this moderating effect, we tap on a recent finding by Ding et al. (2020) that neuroticism moderates the direct effect of trait mindfulness on sleep quality in a university student population. A higher level of trait mindfulness is associated with less sleep disturbance and hence better sleep (Garland et al., 2013). Individuals high in neuroticism, however, tend to pay more attention to negative stimuli which contributes to the maintenance of sleep disturbances (Ding et al., 2020). Thus, neuroticism serves to weaken the positive effect of trait mindfulness on sleep quality. Since neuroticism is consistently found to be associated with lower levels of psychological well-being (Brown and Ryan, 2003), we can expect that as a consequence of a negative experience (resulting in a decrease in affect balance), an individual with poor psychological well-being will have more sleep disturbance compared to one who is high in the same.

### Sleep and Academic Performance

Consistent with the findings of other studies (Barahona-Corea et al., 2018; Maheshwari and Shaukat, 2019), our univariate analysis showed that daytime dysfunction was significantly correlated with CAP scores. Taking CAP to be indicative of academic performance for the semester that our survey was conducted, this result is not surprising since one would expect higher academic achievement with the absence of excessive daytime sleepiness and presence of enthusiasm to study, complete assignments and so on. Due to inconsistencies in the literature concerning the relationship between excessive daytime sleepiness and academic performance (Hangouche et al., 2018), we hypothesize that impaired sleep negatively impacts academic performance more through the consequential loss of enthusiasm to get things done–including academic motivation–than excessive daytime sleepiness. Our univariate analysis, however, did not reveal any association between sleep quantity and the other sleep quality measures with CAP. These results are consistent with the findings of Eliasson et al. (2010), Sweileh et al. (2011), Jalali et al. (2020), and King et al. (2018).

Our multivariate analysis revealed that academic performance is quadratically related to overall sleep quality, and this relationship is moderated by psychological well-being such that at low levels of psychological well-being, this relationship is an inverted-U while at high levels, it is U-shaped. Together, these U-shaped and inverted U-shaped relationships indicate that poor sleepers can have good CAPs and good sleepers can have poor CAPs. Indeed, such students were found within our sample. For example, the top student with the highest CAP among the poor sleepers typically went to bed at 3.30 a.m. and had 4.5 h of sleep out of the total time of 6 h in bed, which translates to a poor sleep efficiency of 75%. With 1.5 h of remaining awake in bed on average each night, that student clearly suffered from pronounced sleep disturbances. Not surprisingly, that student had a severe Global PSQI score of 11. Yet, he or she had a CAP of 4.92 (or 1.72 standard deviations above the mean). In contrast, the student with the lowest CAP among the good sleepers had a good Global PSQI score of 4, but a CAP of 3.63 (or 1.69 standard deviations below the mean).

The interaction plots shown in Figures 2A,B suggest that among CAPT students with low levels of psychological well-being, those with low affect balance will, on average, have substantially poorer overall sleep quality than those with the same CAP score but high affect balance. Among CAPT students with high levels of psychological well-being, however, those with low affect balance only have slightly poorer overall sleep quality compared to those with the same CAP but high affect balance, on average. These observations are congruent with the differing slopes shown in Figure 2C.

The remainder of this subsection serves to provide a speculative explanation for the trends shown in Figures 2A,B. For brevity, we focus on the “middle ground” of the two scenarios depicted, i.e., the case of *mean* affect balance. In this case, the two quadratic curves meet precisely at their turning points. Since the turning points coincide with the mean CAP score, we can speak of four types of students: those with *low* levels of psychological well-being and *below average* CAPs (Type 1) versus *above average* CAPs (Type 2), and those with *high* levels of psychological well-being and *below average* CAPs (Type 3) versus *above average* CAPs (Type 4).

Sutin et al. (2020) summarized that among the Big Five personality traits, lower levels of extraversion and conscientiousness and higher levels of neuroticism are associated with poorer sleep, while openness to experience and agreeableness are not associated consistently with sleep quality. Moreover, Duggan et al. (2014) found that low conscientiousness and high neuroticism are the best predictors of poor sleep. Thus, we expect Type 1 and 2, and Type 3 and 4 students to be higher in neuroticism and conscientiousness, respectively. Hakimi et al. (2011) found that all the Big Five personality traits were related to GPA scores; conscientiousness was positively correlated and was the strongest predictor, followed by neuroticism which was negatively correlated.

Next, we turn to the concept of *flow*, known colloquially as being “in the zone”. Flow is a state of deep absorption in an activity wherein the individual functions at her fullest capacity and the experience itself is intrinsically rewarding such that the individual seeks to replicate it (Shernoff et al., 2003). Scholars have reported experiencing flow when engaged in their best work (Csikszentmihalyi, 1996). Hager (2015) concluded that individuals higher in extraversion, openness and conscientiousness have a greater disposition to experience flow, while high neuroticism hinders an individual from having such experiences. We therefore expect that Type 3 and 4 students have more flow experiences than Type 1 and 2 students while engaged in learning activities.

Sumaya and Darling (2018) reported that students who experienced flow while working on a particular assignment scored significantly higher grades than students who did not. Ozhan and Kocadere (2019) concluded that flow increases academic success through increased motivation. We therefore hypothesize that for Type 3 and 4 students, conscientiousness provides the initial motivation to improve their CAPs, which is subsequently strengthened by flow experiences. We further posit that the increased motivation leads Type 3 students to engage in longer night-time study to the detriment of their sleep. Type 4 students on the other hand, are better able to replicate flow experiences and leverage these experiences to maximize their learning efficiency. As a result, these students are able to improve their CAPs without negatively impacting their sleep.

Turning to Type 1 and 2 students, Komarraju et al. (2009) suggested that anxiety provides students high in neuroticism the motivation to do better. Bratko et al. (2006) similarly suggested that anxiety and perfectionism in such students could lead to improved academic performance. On this note, we hypothesize that anxiety provides the motivation for Type 1 students to do better while both anxiety and perfectionism motivate Type 2 students. As the CAPs of Type 1 students improve, their level of anxiety falls, leading to improved sleep. For Type 2 students, however, we speculate that improved CAPs further fuel their desire for “perfect grades”, leading to increased anxiety and in turn poorer sleep.

### Well-Being and Academic Performance

The significant interaction between psychological well-being and CAP scores that our multivariate analysis revealed is consistent with observations made by Richter (2015) that students with higher GPAs had increased levels of certain facets of psychological well-being.

Both simple linear regression and polynomial regression (involving up to third order terms) would, however, fail to detect any association between psychological well-being and CAP scores. In fact, both the Spearman and Kendall rank correlation coefficient would also fail in this regard. This suggests that academic performance, as measured by CAP, does not have a direct effect on psychological well-being. For the same reasons, academic performance does not appear to have a direct effect on the affective components of subjective well-being as well. Therefore, contrary to what one might expect, well-being cannot mediate the relationship between academic performance and overall sleep quality. Academic performance can possibly only moderate the relationship between the affective components of subjective well-being, and psychological well-being. The present study, however, did not examine academic performance in such a role.

### Comparisons With Other Student Populations

Based on the results in Section “Global PSQI, SPANE, and FS Scores of Other Student Populations”, we conclude that CAPT students have lower affective well-being compared to their counterparts in America, Canada, Germany, Portugal, and South Africa, but similar affective well-being compared to students in Japan and SMU. For FS scores, only the differences in the mean between CAPT and SMU students are not significant. Thus, CAPT students apparently have lower psychological well-being compared to their overseas counterparts with the exception of students in Japan who are not doing as well in this regard. On the other hand, CAPT and SMU students have comparable psychological well-being. We also conclude that CAPT students have poorer overall sleep quality compared to their counterparts in United States, Belgium, Taiwan, and Nigeria, similar overall sleep quality compared to students in Brazil, Luxembourg, and Germany, and better overall sleep quality compared to students in Indonesia.

### Limitations and Future Directions

The present study has several key limitations. First, a wider sample would have allowed for more robust results to be generated. Secondly, the availability of additional pertinent data would have enabled a better model for overall sleep quality to be obtained and more in-depth analyses to be made. This includes student demographics such as gender and year of study, and other sleep-related information such as frequency and duration of daytime naps, chronotype, consistency of sleeping patterns, as well as objective sleep measures (e.g., actigraphy-based recordings). Greater sleep consistency has been linked to better academic performance and may have a greater impact on GPA than sleep duration (Hershner, 2020). Napping has been shown to improve logical reasoning and moods, reduce subjective levels of daytime sleepiness (Milner and Cote, 2009), and improve overall sleep quality (Wang and Biro, 2021). Chronotype preference toward eveningness has been found to be associated with poorer academic performance (Gomes et al., 2011; Hershner, 2020). Thirdly, the inclusion of the Satisfaction with Life Scale by Diener et al. (1985) would have provided a more complete view of the levels of subjective well-being of our subjects. Fourthly, we believe that the inclusion of an instrument to assess the Big Five personality traits, e.g., the Big Five Inventory (John and Srivastava, 1999), would have improved the quality of fit of our model, given the growing evidence of the influence of personality traits on subjective sleep (Sutin et al., 2020). Finally, CAP is a partial measure of academic performance and achievement. Complementing it with other quantitative and qualitative measures would have provided more extensive information about the academic experience of the participants.

A future direction would be to develop separate models for objective and subjective sleep quality, taking into account the above limitations. This would allow for an in-depth investigation into the direct and indirect effects that academic achievement, well-being, personality traits and other individual differences such as chronotype preference, have on subjective and objective sleep quality. As it has been recognized that sleep and well-being affect each other (Steptoe et al., 2008; Lau et al., 2015; Zou et al., 2020), we would also include in the proposed study, an investigation into the effects of subjective and objective sleep quality on well-being. Libman et al. (2016) pointed out that sleep quality is a construct often measured but never defined. Ness and Saksvik-Lehouillier (2018) similarly stated that sleep quality “seems to lack an established definition”. Taking the cue from these authors, a key objective of the proposed study would be to determine the extent to which the proposed two models concur, in the pursuit of establishing a holistic understanding of what it means to have quality sleep, both objectively and subjectively, as well as its antecedents and consequences. Libman et al. (2016) and Harvey et al. (2008) have made a start in this direction with their findings that the daytime experience of feeling refreshed versus nonrefreshed in the morning, and the night-time experience of good versus impaired sleep continuity, characterizes perceived good versus poor sleep. Nevertheless, that still leaves much to be desired in our opinion.

## Data Availability Statement

The raw data supporting the conclusions of this article will be made available by the authors, without undue reservation.

## Ethics Statement

The studies involving human participants were reviewed and approved by Institutional Review Board, National University of Singapore. The patients/participants provided their written informed consent to participate in this study.

## Author Contributions

AC and FB conceptualized the present study. FB took charge of the well-being instruments to be used, helped to refine sections “Introduction”, “Instruments” and contributed to section “Procedure,” the preamble of section “Discussion,” “Sleep and Psychological Well-Being,” and the discussion on limitations in section “Limitations and Future Directions.” MA processed the raw data, performed the statistical analyses, and wrote all sections of the manuscript including developing all the hypotheses and speculations in section “Discussion.” AC wrote the background of the college at the start of section “Participants and Bad Data Points” and contributed to section “Procedure”. AC suggested in part for the section “Global PSQI, SPANE, and FS Scores of Other Student Populations.” All authors contributed to the article and approved the submitted version.

## Conflict of Interest

The authors declare that the research was conducted in the absence of any commercial or financial relationships that could be construed as a potential conflict of interest.
